# Automation in food analysis

**DOI:** 10.1155/S1463924681000291

**Published:** 1981

**Authors:** Clive J. Jackson


wound in a double helix. Fluid transfer power is supplied by compressed air, whilst the valving system is exposed solely to "clean" fluids – air and methylene chloride. Use of an appropriate phase separator permits other water/solvent combinations to be used.

Massie [15] has developed a multi-channel, continuous flow instrument for use in water testing laboratories. This instrument is capable of running six tests simultaneously, many of the tests in the part-per-billion range. The automatic sampler has a special feature so that it can accommodate samples with different matrices, such as acid digested samples for a TKN determination. The programmer is capable of interfacing with two samplers; therefore, four different sample matrices can be handled at one time.

Conetta [16] presented a technique whereby total phosphate in water samples may be determined by a photochemical decomposition of organic phosphorus compounds and the thermal hydrolysis of acid-hydrolysable phosphates followed by the conventional colorimetric determination of the liberated ortho-phosphate with molybdenum blue. Analytical performance data were presented and discussed.

Further details of these papers can be obtained by corresponding with the primary authors. Many of these have indicated that they will be submitting their manuscripts to this Journal for formal publication.

*P.B. Stockwell*

REFERENCES

[1] A continuous in-process surface tension measuring device. *V.P. Janule,* Madison-Kipp Corporation, 201 Waubesa Street, Madison, Wisconsin 53704.

[2] Automation in monitoring gases and particulates in the pulp and paper industry. *T.L.C. De Souza,* Pump & Paper Research Institute of Canada (PPRIC), 570 St. John's Blvd., Pointe Claire, Que., Canada.

[3] Continuous analysis of oxygen in coke oven gas. *Dan P. Manka,* Consultant, 1109 Lancaster Avenue, Pittsburgh, Pennsylvania 15218.

[4] Automated instrumentation for chemical analyses in process control. *H.V. Malmstadt,* School of Chemical Sciences, Univ. of Illinois, Urbana, Illinois 96740.

[5] The changing face of laboratory automation: Present & Future. *P.B. Stockwell,* Editor, Journal of Automatic Chemistry, United Trade Press, 33-35 Bowling Green Lane, London, EC1R 0DA.

[6] Non-specific methods of analysis: the unsung heroes of the process control world. *R.T. Oliver,* Foxboro Analytical, Balsbough Electro-Chemical Ctr., Armstrong Road, Plymouth Industrial Park, Plymouth, MA 02360.

[7] A microprocessor-based control and data reduction system for Multi-channel flow injection analysers. *Dorothea H. Jirauch,* Lachat Chemicals Inc., 10500 N. Port Washington Road, Mequeon. WI 53092.

[8] Simplex optimisation applied to flow injection analysis. *Craig R. Ranger,* Lachat Chemicals Inc., Instrument Division, 10500 N. Port Washington Road, Mequon. WI 53092.

[9] Applications of a new, automated solution handling system utilising flow injection methodology. *K.G. Schick,* Fiatron Systems Inc., 6651 N. Sidney Pl., Milwaukee WI 53209.

[10] Guidelines for designing flow injection analysis systems. *Joseph T. Vanderslice,* USDA, Nutrient Composition Lab., Room 225, Building 308, Barc-East, Beltsville, MD 20705.

[11] A model for two phase AMFIA systems. *Kent T. Stewart,* USDA, Nutrient Composition Lab., Room 225, Building 308, Barc-East, Beltsville, MD 20705.

[12] Use of an exponential dilution chamber as a means of scale expansion in flow injection analysis (FIA). *Kent K. Stewart,* USDA, Nutrient Composition Lab., Room 225, Building 308, Barc-East, Beltsville, MD 20705.

[13] Some aspects of automated sample preparation. *Robert Weinberger,* Technicon Industrial Systems, Tarrytown, New York 10591.

[14] A novel, automated liquid extraction technique for aqueous pesticides samples. *P. Baulu,* Pesticides Section, Laboratory Services Branch, Ministry of the Environment, PO Box 213, Rexdale, Ontario, Canada.

[15] A new continuous-flow, highly sensitive, six channel water analyser. *Lynne Massie,* ALPKEM Corporation, PO Box 1260, Clackamas OR 97015.

[16] An automated method for the measurement of total phosphate by UV digestion. *A. Conetta,* Technicon Industrial Systems, Tarrytown, New York 10591.

### Automation in food analysis

A joint meeting on the above topic, organised by the Automatic Methods Group and South East Region of the Analytical Division of the Royal Society of Chemistry, UK, was held in Leatherhead, Surrey on 12 December 1980.

In the morning session Dr Folkes [1] discussed the applications of HPLC in the analysis of foodstuffs; he particularly emphasised the contribution of automation both during the development of analytical procedures and subsequently during their routine use. The second paper was presented by Mr Coverly [2] who discussed the automation of on-line sample preparation procedures for HPLC, with particular reference to solid-liquid extraction, liquid-liquid extraction, concentration, solvent exchange and derivatisation techniques. The potential for the incorporation of these procedures into fully automated analytical systems for use in the food industry was considered. The final paper of the morning session was given by Dr Saxby [3] who briefly described a compact computer-controlled quadrupole mass spectrometer coupled to a gas chromatograph. The application of the technique was illustrated by reference to work on the detection of taints and off-flavours in foodstuffs. The use of the peak-finder mode of operation, in which mass spectra are recorded on all peaks from the GC, was exemplified by reference to the detection of chlorobenzene in milk products. The use of ion-monitor mode, in which only selected ions are monitored, was illustrated by the detection of chloroanisoles, chlorophenols and mesityl oxide in a variety of materials.

After lunch the Automatic Methods Group held a short AGM before the first paper in the afternoon session in which Dr Osborne [4] discussed the application of near IR(NIR) in the automated analysis of protein and moisture levels in cereals and cereal products. He also indicated further possible applications including the measurement of flour colour, degree of starch damage and the prediction of bread-making quality of the flour. The second paper, by Mr Davies [5] considered the use of automated ion-selective electrode analytical procedures with particular reference to the determination of trace chloride levels in poultry meat. Finally, Mr Steele [6] described some automated on-line monitoring systems for the measurement of a variety of physical parameters of food materials during processing. He particularly emphasised the impact of micro-electronics in the fields of weighing, sorting, flow measurement and sizing, and the contribution of microprocessors in the field of integrated plant control systems. Finally he gave a brief insight into the future with a mention of the use of a microprocessor controlled automated NMR spectrometer for the measurement of fat in a chocolate ingredient.

The meeting was well received by the audience and a particularly useful exchange of information and ideas was obtained during the two discussion periods at the end of the morning and afternoon sessions. It was unfortunate that an attendance of 35 did not do justice to the excellent contributions from the speakers and the superb facilities provided by our hosts BFMIRA.

*Clive J. Jackson*

*See page 103 for references*

REFERENCES
[1] The use of automation in the development and application of chromatographic methods in food analysis. Dr D. Folkes, RHM Central Research Laboratory, High Wycombe, Bucks.
[2] Automated sample preparation for HPLC-techniques, applications and some possibilities in food analysis. S. C. Coverly, Technicon Instrument Co. Ltd., Basingstoke, Hants.
[3] Applications of GC/quadrupole MS in trace food analysis, Dr. M. J. Saxby, BFMIRA, Leatherhead, Surrey.
[4] Application of near IR to the analysis of food. Dr. B.G. Osborne, Flour Milling and Baking Research Association, Chorleywood.
[5] Determinaiton of trace chloride in poultry meat using automated ion - selective electrode techniques. A.M.C. Davies, Food Research Institute, Norwich.
[6] Process instrumentation in the food factory. D.J. Steele, BFMIRA, Leatherhead, Surrey.

### Product News

### Atlantic City revisited 1981

*A review of exhibits at this year's Pittsburgh Conference*

The 32nd Annual Pittsburgh Conference on Analytical Chemistry and Applied Spectroscopy was held from 9 - 13th March at Atlantic City, NJ, USA (A report on the scientific meetings appears on page 100). In true tradition it was bigger, better attended and had more lectures presented at it than before. As a regular visitor one was left with the opinion that perhaps there is really very little new under the sun. Microprocessors were first presented several years ago and now they are mostly common place, while visual display units and graphics displays increase in numbers and sophistication. This year there was a considerable interest in computer systems and networking.

The show presented the latest state of the art instrumentation, and these instruments are more and more sophisticated – however, do they fulfill the true aims of the analyst? Very often it is sad to note that instrument companies have developed their systems without regard to the needs of the analyst; particularly they do not often allow facilities for filing data and interogation of the data at a future date.

It is impossible to cover all the aspects of instrumentation on show which related to automation, however the few which are discussed here typify those on show.

### Spectrophotometer

The new DU-5 UV-visible/NIR computing spectrophotometer displayed by Beckman automates procedures in analysing aerosols, polymers, paints, food, drugs, water and biologicals. This table-top instrument includes a spectrophotometer, microcomputer and printer in one unit with software memory storage modules. It can be programmed for specific user applications with quick change-over between analyses. The DU-5 stores internally three individual analysis programs. Memory-Pac plug-in software modules offer program storage for five additional analyses with an EA-ROM non-volatile memory that will not erase in a power failure or when removed from the instrument.

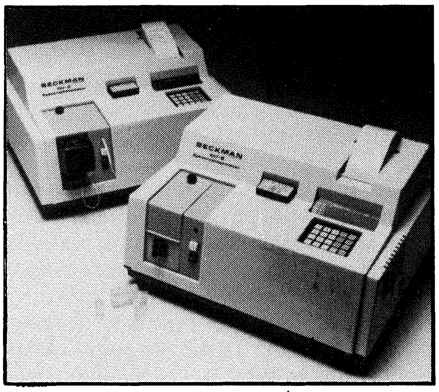

*Beckman's DU-5 UV visible/NIR computing spectrophotometer.*

*Beckman Instruments Inc., 2500 Harbor Boulevard, Box 3100, Fullerton, Cal. 92634, USA.*

### Atomic fluorescence spectrometer

The Baird Corporation introduced their Plasma/AFS which is currently the only commercially available atomic fluorescence spectrometer. Intended for the simultaneous determination of any 12 of 65 different elements, it delivers up to 1,800 determinations per hour and offers a cost-effective alternative to traditional atomic absorption and plasma emission techniques. It has a linear dynamic range of 4 - 5 orders of magnitude with "virtually no spectral interferences". Setup for a different combination of elements is fast and requires no optical or mechnical alignment.

*Baird Corporation, 125 Middlesex Turnpike, Bedford, MS 01730, USA.*

### Computer interface

UTI has developed a microprocessor-based interface which links their range of mass spectrometers with virtually any computer on the market. The Spectra-Link is compatible with the IEEE 488, RS-232C and RS-449 interface standards. The unit includes software stored in read-only memory (firmware) for five operating modes, Four of these (spectrum scan mode, total pressure mode, specific peak mode, and calibration mode) automatically perform most of the common tasks required to control the unit. The fifth, direct control mode, permits even more operator flexibility by allowing the user to write totally original programs.

*UII, 325 N. Mathilda Avenue, Sunnyvale, CA 94086, USA.*

### IR Spectrophotometers

Perkin Elmer's new product line of low cost infrared spectrophotometers, the Model 1300 series, was exhibited. There are three models; the 1310 has a single slit program and two scan speeds, the 1320 and 1330 have three scan speeds and two appropriate slit programs. The series can be interfaced to the Model 3500 infrared data station providing an improvement of spectral quality and the facility to use the spectrophotometer for 'Search' applications in the identification of unknowns.

*Perkin-Elmer Corp, Main Avenue (MS-12), Norwalk, CT 06856, USA.*

**Editor's Note:**· *The address of the manufacturer/supplier appears in italics at the end of each item. In some cases this address will be that of a subsidiary to the manufacturing company as the address given is that from which the information has been obtained.*

